# Evolutionary history and climate-driven dynamics of transposable elements has shaped genome evolution in the *Coffea* genus

**DOI:** 10.1038/s41598-026-40031-6

**Published:** 2026-02-18

**Authors:** Mathilde Dupeyron, Laura Gonzalez-Garcia, Simon Orozco-Arias, Rickarlos Bezandry, Nathalie Raharimalala, Luiz Felipe Protasio Pereira, Dominique Crouzillat, Petra De Block, Coralie Fournier, Laurence Bellanger, Patrick Descombes, Perla Hamon, Douglas Silva Domingues, Romain Guyot

**Affiliations:** 1 Institut de Recherche pour le Développement (IRD), UMR DIADE, Université de Montpellier, CIRAD, Montpellier, France; 2 Boyce Thompson Institute, Ithaca, NY USA; 3 Department of Computer Science, Universidad Autónoma de Manizales, Manizales, Colombia; 4 Life Sciences Department, Barcelona Supercomputing Center, Barcelona, 08034 Spain; 5 Faculté des Sciences, de Technologies et de l’Environnement (FSTE), Université de Mahajanga, Campus Universitaire d’Ambondrona, BP 652, Mahajanga, 401 Madagascar; 6 Ecole Doctorale Ecosystèmes Naturels (EDEN), Université de Mahajanga, Mahajanga, Madagascar; 7 Centre National de Recherche Appliquée au Développement Rural, BP 1444, Ambatobe, Antananarivo, 101 Madagascar; 8 Laboratório de Biotecnologia Vegetal, Instituto de Desenvolvimento Rural do Paraná—IAPAR-EMATER, Londrina, CEP 86047-902 PR Brazil; 9 Embrapa Café, Brasília, CEP 70770-901 DF Brazil; 10 12, chemin de la Gaspière, Cerelles, 37390 France; 11 Meise Botanic Garden, Meise, Belgium; 12 Société des Produits Nestlé SA, Nestlé Research, Lausanne, Switzerland; 13 Société des Produits Nestlé SA, Nestlé Research, Tours, France; 14 Department of Genetics, “Luiz de Queiroz” College of Agriculture, University of São Paulo, ESALQ/USP, Piracicaba, Brazil; 15 Department of Electronics and Automation, Universidad Autónoma de Manizales, Manizales, Colombia; 16 Present Address: Hopitaux Universitaires de Genève, Campus Biotech, Geneva, 1202 Switzerland

**Keywords:** Ecology, Ecology, Evolution, Genetics, Plant sciences

## Abstract

Genome size variation is a fundamental feature of plant genomes and plays an important role in phenotypic diversity, ecological adaptation, and plant evolution across angiosperms. In the *Coffea* genus (*Rubiaceae*, 141 species/taxa), significant genome size variations have been observed. There has been nearly a twofold increase between species from East and West Africa and a notable increase from northwest to southeast Madagascar, resulting in geographic gradients. Previous studies suggest a role of Long Terminal Repeat (LTR) retrotransposons in these variations; however, the low resolution of the data to support this hypothesis did not allow for a clear understanding of LTR retrotransposons dynamics within the genus. Here, we present an analysis of the genomes of 22 *Coffea* species mainly from Africa and Madagascar and their genome size variations within a robust phylogenetic framework. Our results show that genome size and Transposable Elements (TE) landscape are first structured by phylogenetic relationships, reflecting shared evolutionary history and lineage-specific LTR retrotransposon dynamics particularly involving the Tekay/Del, TAT, and SIRE lineages. These lineages contribute to the differentiation of phylogeographic groups, reflecting specific patterns of genomic divergence linked to species adaptation and speciation. We also detected significant association between specific TE families and environmental variables (such as isothermality and annual precipitation). These correlations suggest that environmental factors modulate repeatome evolution and a potential adaptive role of these TEs. These findings highlight the importance of TEs in genome dynamics at the intersection of evolutionary processes and environmental adaptations and open new perspectives on their adaptive role within the *Coffea* genus.

**Supplementary Information:**

The online version contains supplementary material available at 10.1038/s41598-026-40031-6.

## Introduction

Plant genomes provide a remarkable example of how evolution shapes biological diversity. Genome size variation is a particularly intriguing phenomenon, with sizes differing by more than 2440-fold in land plants^1^. In plants, Transposable Elements (TEs) are the primary drivers of genome size variation, beside polyploidization, accounting for between 3 and 85% of total genomic sequences^2,3^. These repetitive sequences, collectively referred to as the repeatome, comprise not only TEs but also tandem repeats and other repeat families. The repeatome represents a dynamic component of genomes, influencing their composition, architectures, regulations and evolution.

Long Terminal Repeat (LTR) retrotransposons are frequently associated with genome size changes due to their replication mode, which involves an RNA intermediate, a process known as “copy-and-paste” transposition^4^. These amplification events, or “bursts” can have dramatic consequences on chromosome structure, as illustrated in the genome of the wild rice *Oryza australiensis* (Poaceae, order Poales)^5^. In this species, a rapid genome size doubling was observed due to the massive activity of only four LTR retrotransposon families. Besides these families, LTR retrotransposons alone make up 61% of this diploid genome’s sequences^6^ and contribute to a clear genomic differentiation from cultivated Asian rice (*Oryza sativa*).

The amplification of TEs can induce chromosomal rearrangements, including fragment losses, inversions, duplications, and translocations, which are also frequently observed in plant genomes that have undergone polyploidization events^7^. Genomic differentiation driven by transposable element mobilization can also lead to the emergence of genetic incompatibilities, ultimately resulting in reproductive barriers and eventually speciation^8,9^. Changes in LTR retrotransposon activity have been observed during the diversification process of *Citrus* species (Rutaceae, order Sapindales) contributing to the formation of modern species^10^. Similarly, the independent expansion of LTR retrotransposons and their divergent evolutionary trajectories are thought to have contributed to the speciation process in *Oryza* species^11^. Thus, variations in the repeatome may impact multiple aspects of species evolution, particularly genomic divergence and speciation processes.

LTR retrotransposons can respond to biotic and abiotic stresses, inducing their activity and leading to their accumulation in genomes^12^. These observations have led to the hypothesis that TEs can play a key role in species’ environmental adaptation, particularly during abrupt environmental changes^13–15^. To understand the role of TEs in adaptation, some studies have investigated the relationship between TE dynamics and ecological factors in non-model plant species. A recent study showed that the genome size in palm trees (Arecaceae, order Arecales) is constrained by climatic factors (such as aridity), suggesting that water stress may inhibit the activation of certain TE families^16^. A similar trend has also been observed in wild *Coffea* species from Madagascar, where a correlation between humid environments and larger genome sizes was reported^17^.

The *Coffea* genus (family *Rubiaceae*) currently comprises 141 species/taxa of tropical trees^18,19^, following the poorly supported inclusion of the *Psilanthus* genus into *Coffea*^19^. For this reason and for more clarity for the readers, it is more reasonable to maintain a dual nomenclature for these species (i.e. *Coffea/* ex *Psilanthus*).

**Fig. 1:**
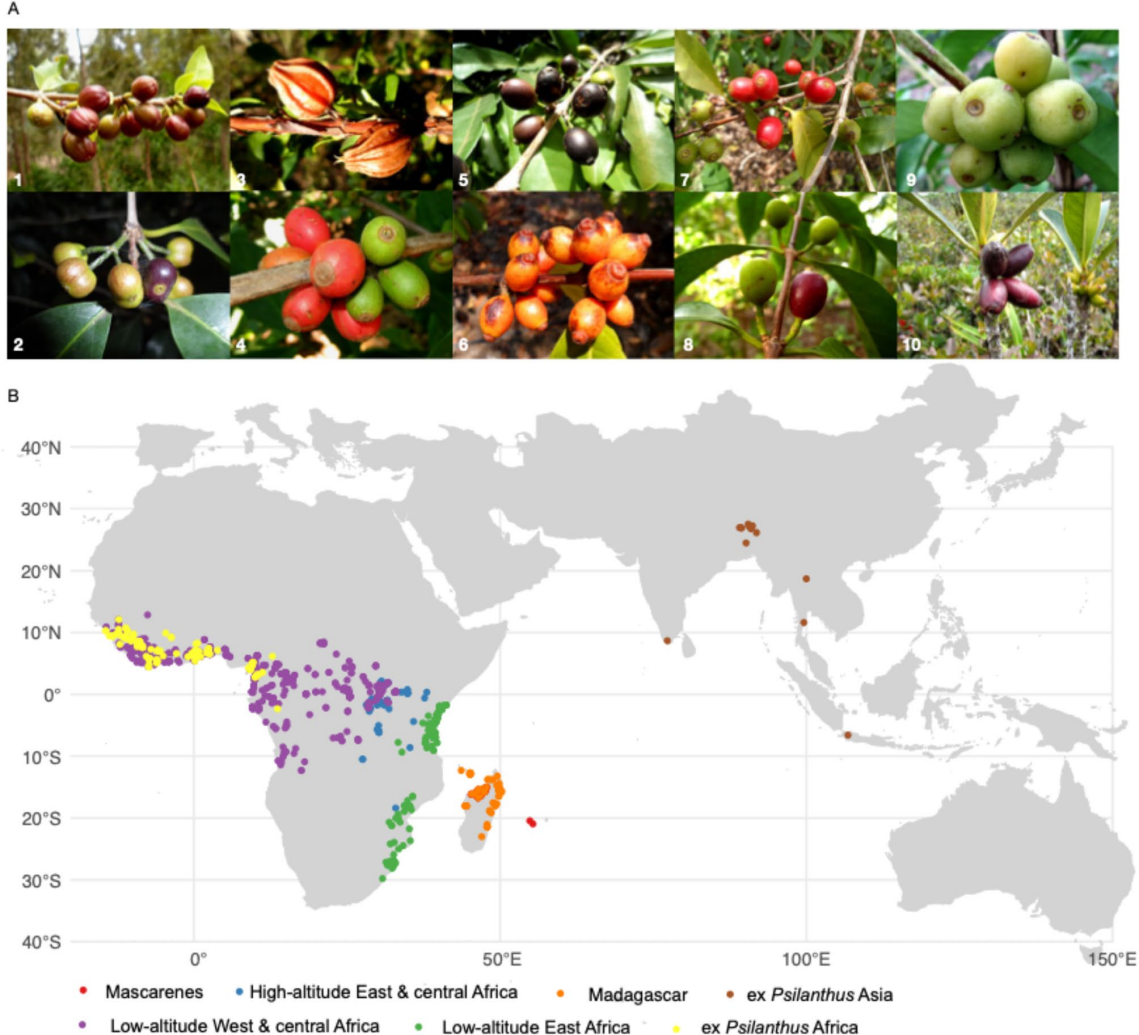
Species diversity and distribution of phylogeographic groups in *Coffea*. (**A**) Diversity of wild *Coffea* species across different phylogeographic groups: (1) *Coffea racemosa*, and (2) *Coffea salvatrix* (Low-altitude East Africa), (3) *Coffea kapakata*, (4) *Coffea liberica*, (5) *Coffea stenophylla*, and (6) *Coffea sp. Congo* (Low-altitude West and Central Africa), (7) *Coffea eugenioides* (High-altitude East and Central Africa), (8) *Coffea humblotiana* and (9) *Coffea dolichophylla* (synonym of *C. millotii*, Madagascar), (10) *Coffea macrocarpa* (Mascarenes). Photo credit: Emmanuel Couturon (IRD, Pictures 1–8 and 10), Nathalie Raharimalala (FOFIFA, Picture), available at 10.23708/JZA8I2. (**B**) Geographic distribution of the studied species by phylogeographic groups. The map was created using the R package *rnaturalearth* (V.1.2.0).

The most well-known species are *Coffea arabica* and *C. canephora*, the two cultivated species that produce coffee beans for global consumption. Beyond these two cultivated species, there are 139 wild species/taxa native to Africa (*Coffea* and ex *Psilanthus*), Madagascar (*Coffea*), Mascarene Islands (*Coffea*), and Asia - Australasia (ex *Psilanthus*)^18^ (wildcoffeedb.org). The highest species diversity is found in Madagascar and the Indian Ocean islands, with at least 66 species. The wild *Coffea* species exhibit exceptional phenotypic diversity^20,21^ (Fig. 1) and remarkable environmental adaptations occupying highly contrasting ecological niches, from dry savannas (*Coffea neoleroyi*/ex *Psilanthus leroyi*) to humid montane tropical forests (*Coffea kivuensis*). Some *Coffea* species exhibit remarkable ecological flexibility, adapting to both dry and humid habitats, such as *Coffea ebracteolata (*ex *Psilanthus ebracteolatus)*, *C. canephora* and *C. liberica*. Additionally, certain Malagasy species, such as those belonging to the Baracoffea group, demonstrate exceptional tolerance to water deficit and high temperatures^17^.

The phylogeny of the *Coffea* genus (including the analysis of 80 species) was fully resolved in 2017 using a high-throughput approach and the identification of 28,800 nuclear SNPs^22^. A geographical differentiation of *Coffea* genomes has been identified, forming major phylogeographic groups: (i) low-altitude species from West and Central Africa, (ii) high-altitude species from Central and East Africa, (iii) low-altitude species from East Africa, (iv) species from Madagascar, (v) species from the Mascarene Islands, and (vi) species from the former *Psilanthus* genus in Africa and (vii) in Asia (Fig. 1).

Genetically, all *Coffea* species are diploid (2n = 22), except for *Coffea arabica*, which is an allotetraploid resulting from the hybridization of two wild species (*C. canephora* and *C. eugenioides*) approximately 600,000 years ago^23,24^. In the *Coffea* genus, diploid genome sizes range from 463 Mb for *C. mauritiana*^25^ to 887 Mb for *C. humilis*^26^, with a gradient of increasing genome size from East to West Africa and from the northwest to the southeast in the Indian Ocean islands. An exception is observed in the allotetraploid genome of *Coffea arabica*, which has an estimated size of approximately 1.2 Gb^24^. To investigate the relationship between genome size, TE content, and phylogeographic groups, a partial sequencing analysis was performed on eleven *Coffea* species using 454 sequencing technology^27^. The results revealed a correlation between TE content, genome size, and phylogeographic groups. Interestingly, variations were also observed among different accessions of *C. canephora*, the species with the widest geographic distribution and a notable ecological flexibility. However, due to the limited sequencing coverage and the absence of global positioning system (GPS) and climatic data, it was not possible at the time to study the relationship between genome size, environmental adaptation, and evolutionary heritage dictated by phylogeny in the *Coffea* genus. Similarly, the molecular mechanisms underlying TE accumulation and their role in species divergence and speciation remained unexplored.

Here, we aim to explore the respective contributions of evolutionary history and environmental adaptation to genome size variation within the *Coffea* genus. Specifically, we examine how genome size and TE composition vary across phylogeographic groups and environmental gradients, to evaluate the relative influence of phylogenetic constraints and ecological factors on genome evolution. To address this question, we conducted a multidimensional analysis on 22 *Coffea* species representing the seven known phylogeographic groups. First, we examined the phylogenetic context and evolutionary mechanisms underlying genome size variation. Next, we analyzed the dynamics of TEs and their differential roles across phylogeographic groups. Finally, we explored the influence of environmental factors on these genomic variations, discussing their potential adaptive role.

## Materials and methods

### Plant material, DNA extraction, and genome sequencing

Nineteen *Coffea* (*Coffea* and ex *Psilanthus*; Rubiaceae, subfamily Dialypetalanthoideae, tribe Coffeeae) plants from the Centre de Ressource Biologique (CRB) *Coffea* collection (https://www.ibisa.net/annuaire-crb/coffea-296.html) and related resources were selected. The selected species and accessions were chosen because they have been used and published in our previous phylogenetic analyses, and together represent a broad phenotypic and geographical diversity (https://www.wildcoffeedb.org/home, Table 1). All research involving wild *Coffea* species fully complied with regulations. Collection and handling of plant material were performed under appropriate authorizations. No IUCN- or CITES-listed species were collected from the wild. All accessions originate from established ex-situ collections. African species (including *Coffea kapakata*, *C. stenophylla*, *C. eugenioides*, and *C. humblotiana)* were obtained from the CRB Coffea (https://www.ibisa.net/annuaire-crb/coffea-296.html)*.* It derives from prospecting campaigns conducted between 1960 and 1980 (for African species) and between 2000 and 2010 (for Indian Ocean islands species). Malagasy Materials were obtained from FOFIFA Kianjavato and Jardin Botanique de l’Université de Mahajanga, (Madagascar) and the outgroup (*Kraussia floribunda*) was obtained from Meise Botanical Garden, Belgium). So, all plant material was originated from *ex situ* collections where the identification was performed and confirmed by expert committee of each collection. Table 1 indicates the original publications where the leaf material was previously described, analyzed, or previously sequenced and publicly released.

DNA was extracted from young leaves using DNeasy Kit (QIAGEN) following the manufacturer’s instructions and sequenced, following the protocol described in^29^. Short-read sequencing (carried out between 2012 and 2018), was performed using HiSeq2500 (Illumina) to generate 2 × 125 bp paired-end reads. Public genome sequencing data from nine accessions was also downloaded from NCBI, to build a final panel of 23 species analyzed, including an outgroup from the Rubiaceae family (*Kraussia floribunda*; Rubiaceae, subfamily Dialypetalanthoideae, tribe Octotropideae). See the detailed information of accessions in Table 1. Three species (*Coffea canephora*,* C. eugenioides*,* and Coffea benghalensis*/ex *P. benghalensis*) were represented by more than one accession or variety.

**Table 1:** List of the accessions used.

Species name	Plant code	Plant location	Country of origin	Availability of data	Genome size estimation (Mb)^17,25,26,28^	Source of dry leaf material (LM) or DNA sequence (SEQ)
***Coffea***						
*C. canephora* Pierre ex A.Froehner	C021	BRC	Ivory Coast	SRR18336454	747	SEQ Published in 10.1038/s41588-024-01695-w
*C. canephora* Pierre ex A.Froehner	BUD15	BRC	Uganda	SRR18336434	747	SEQ Published in 10.1038/s41588-024-01695-w
*C. canephora* Pierre ex A.Froehner	HD	BRC	Democratic Republique of Congo	SRR18336427	747	SEQ Published in 10.1126/science.1255274
*C. canephora* Pierre ex A.Froehner	C033	BRC	Ivory Coast	SRR18336408	747	SEQ Published in 10.1038/s41588-024-01695-w
*C. congensis* A.Froehner	CC53	BRC	Republique of Congo	ERR15695989 *	743	LM Published in 10.1038/s41598-021-87419-0
*C. dewevrei* De Wild. & T.Durand	EB51	BRC	Centrafrique Republique	ERR15695991 *	678	LM Published in 10.1038/s41598-021-87419-0
*C. dolichophylla* J.-F.Leroy (syn. to *C. millotii*)	DOL/A.206 (P)	KCRS	Madagascar	SRR16074882 *	680	LM Published in 10.1038/s41598-021-87419-0
*C. eugenioides* S.Moore	DA (P)	BRC	Kenya	SRR35106166 *	709	LM Published in 10.1038/s41598-021-87419-0
*C. eugenioides* S.Moore	BUA	–	Uganda	ERR15695990 *	709	SEQ Published in 10.1038/s41588-024-01695-w
*C. humblotiana* Baill.	BM19/20 (K, MO, TAN)	BRC	France	SRR12696857	468	SEQ Published in 10.1038/s41598-021-87419-0
*C. humilis* A.Chev.	G57 (K)	BRC	Ivory Coast	SRR16074873 *	887	LM Published in 10.1038/s41598-021-87419-0
*C. kapakata* (A.Chev.) Bridson	KAP	BRC	Angola	ERR15695992 *	668	LM Published in 10.1038/s41598-021-87419-0
*C. liberica* W.Bull. ex Hiern	EA61	BRC	Ivory Coast	ERR15695993 *	729	LM Published in 10.1038/s41598-021-87419-0
*C. macrocarpa* A.Rich.	PET (P, K)	BRC	Mauritius	SRR16074881 *	564	LM Published in 10.1038/s41598-021-87419-0
*C. pseudozanguebariae* Bridson	H53 (K)	BRC	Kenya	ERR15695994 *	593	LM Published in 10.1038/s41598-021-87419-0
*C. racemosa* Lour.	IB62 (K)	BRC	Mozambique	SRR16074869 *	499	LM Published in 10.1038/s41598-021-87419-0
*C. salvatrix* Swynn. & Philipson	C408FL	BRC	–	ERR15695995 *	589	LM Published in 10.1038/s41598-021-87419-0
*C. sessiliflora* Bridson	PA60	BRC	Tanzania	ERR15695996 *	535	LM Published in 10.1038/s41598-021-87419-0
*C. sp.* ‘Congo’	C416FL	BRC	Republique of Congo	ERR15695997 *	651	LM Published in 10.1038/s41598-021-87419-0
*C. stenophylla* G.Don.	FB55 (K)	BRC	Ivory Coast	SRR16074866 *	620	LM Published in 10.1038/s41598-021-87419-0
*C. tetragona* Jum. & H.Perrier	A.252 (K, MO, TAN)	KCRS	Madagascar	SRR16074865 *	516	LM Published in 10.1038/s41598-021-87419-0
*C. ambongensis*	BR071	UM	Madagascar	SRR22329145	567	SEQ Published in 10.1371/journal.pone.0296362
*C. bissetiae*	BR03	UM	Madagascar	SRR22329144	581	SEQ Published in 10.1371/journal.pone.0296362
*C. boinensis*	BR051	UM	Madagascar	SRR22329143	562	SEQ Published in 10.1371/journal.pone.0296362
***ex Psilanthus***						
*P. benghalensis var. bababudanii* (Sivar., Biju & P.Mathew) A.P.Davis	PBT1	CBI	India	SRR16074880 *	709	LM Published in 10.1038/s41598-021-87419-0
*P. benghalensis* (Heyne ex J.A.Schult.) J.-F. Leroy	PBT5	CBI	India	SRR16074864 *	709	LM Published in 10.1038/s41598-021-87419-0
*P. ebracteolatus* Hiern	PSI11 (K, P)	BRC	Ivory Coast	SRR16074879 *	550	LM Published in 10.1038/s41598-021-87419-0
***Outgroup***						
*Kraussia floribunda* Harv.	Kra-Flo-63	BR	Southeastern Africa	SRR18733958	539	–

Germplasm source: BR (Meise botanic garden, Belgium), BRC (Biological resources Center, Reunion), CBI (Coffee board of India), KCRS (Kianjavato coffee research Station, Madagascar), UM (Botanical garden of the university of Mahajanga, Madagascar). The asterisks (*) indicate new sequencing data.

### Phylogenetic analysis and molecular dating

We used short read sequencing data to reconstruct the phylogenetic relationships of the analyzed species, with *Kraussia floribunda* as the outgroup. The estimated sequencing coverage per sample ranged from 11× to 97× for *Coffea* species and exceeded 200× for *Kraussia floribunda*. Reads were mapped against the reference genome of *Coffea canephora* (accession HD200^30^), following the strictly identical approach to that described in^22^. Results were filtered to keep Single Nucleotide Polymorphisms (SNP) corresponding to our database of 28,800 SNPs previously described^22^ and heterozygocity was coded using the IUPAC nucleotide code. The concatenated SNPs were then aligned using MAFFT (V. 7.525)^31^, and a phylogenetic tree was generated using FastTree (V. 2.1.10)^32^ and RAxML (V. 8.2.12)^33^. Divergence times were estimated using the divergence age between the ex *Psilanthus* species and *Coffea* as a calibration point, as indicated in^34^, following a strictly identical methodology^22^. Genome size data for each analyzed species were retrieved from the wildcoffedb.org. These genome size estimates are derived from multiple previous studies, including^17,25,26,28^. The reconstruction of ancestral genome size states was performed using the R packages *ape* (V. 5.8–1) and *phytools* (V. 2.4–4), while the analysis of phylogenetic signal was conducted using the R packages *ape* (V. 5.8–1), *phytools* (V. 2.4–4), and *Geiger* (V. 2.0.11).

### Identification and analysis of repeats

The most abundant repeats were analyzed in all dataset using the Galaxy platform of the REPEATEXPLORER2 using the comparative analysis pipeline^35^. A total of 50,000 random paired-end sequences were selected for each accession, representing between 1.12 and 2% of the genome size, and analyzed using default parameters. A comparative analysis was then performed for the 23 species (22 *Coffea*, 28 accessions and one outgroup), incorporating genome size data from the references provided for each clade (Table 1), according to the REPEATEXPLORER2 protocol. Repeat analysis was conducted using R scripts to examine the correlation between repeat abundance and genome size. A Principal Component Analysis (PCA) was performed to assess repeat abundance by lineage and the phylogeographic groups of *Coffea* species. The following R packages were used: *ggplot2* (V. 4.0.1), *FactoMineR* (V. 2.11), *factoextra* (V. 1.0.7), and *scales* (V. 1.4.0) for visualization and PCA analysis. To test whether the phylogeographic groups represent statistically distinct clusters based on the TE reads identified by REPEATEXPLORER2, we applied a Permutation Multivariate Analysis of Variance (PERMANOVA) with 999 permutations, implemented in the R package *vegan* (V. 2.6–10), and a Redundancy Analysis (RDA), also performed in *vegan*. Finally, to determine whether there were significant differences in repeat composition among phylogeographic groups a Kruskal-Wallis test was conducted with the R package *vegan* (V. 2.6–10). Correlation analyses were conducted using the *reshape2* (V. 1.4.4) package in R. Finally, Phylogenetic Generalized Least Squares (PGLS) methodology was applied to analyze relationships between traits (Genome size, SIRE, Tekay and TAT counts) and the evolutionary history of the *Coffea* genus. PGLS analyses were conducted using the *ape* (V. 5.8–1), *ggplot2* (V. 4.0.1) and *caper* (V. 1.0.3) packages in R.

### Preliminary genome assembly, extraction of reverse transcriptase domains from LTR retrotransposons, and phylogenetic analysis

Short reads from the different accessions were used to generate a preliminary genome assembly using MaSuRCA v. 4.0^36^. The resulting assemblies were then utilized to identify the Reverse Transcriptase (RT) protein domains, using GeneWise (https://www.ebi.ac.uk/%7Ebirney/wise2/). Recovered sequences were aligned using MAFFT (https://mafft.cbrc.jp/alignment/software/), and FastTree was used to infer approximately-maximum-likelihood phylogenetic trees as described in^24^. A phylogenetic tree was constructed using the amino-acid RT domain sequences extracted from the assemblies, with reference sequences from the RexDB database^37^. The trees were then visualized and edited using the following R packages: *ggtree* (V. 3.12.0), *phangorn* (V 2.12.1), *ape* (V. 5.8–1), and *dplyr* (V. 1.1.4). The draft genome assemblies are available on Zenodo (10.5281/zenodo.17311662).

### Association between genome size, transposable elements and environment

Historical climatic data from WorldClim^38^ (19 bioclimatic variables) and elevation data were downloaded at a 30-arc second resolution (https://www.worldclim.org/, version 2.1 climate data for 1970–2000 released in January 2020).

The geographical positions (GPS coordinates) of *Coffea* species were retrieved from GBIF (https://www.gbif.org/) with careful verification and filtering to remove irrelevant records (e.g., *ex-situ* collection locations). A total of 799 occurrences were retained for 18 *Coffea* species (out of 22 initially analyzed) (Supplementary Data 1). Annual historical Bioclimatic data and elevation data were extracted in a custom python script using the *raster* function. A Pearson correlation analysis was conducted between these environmental variables and the repeat content obtained from REPEATEXPLORER2, using a custom R script with the *pheatmap* (V 1.0.12) package. All scripts and supplementary files are publicly available on GitHub https://github.com/rg-ird/TE_evolutionary_dynamics/).

## Results

### Genome size variation in the genus *Coffea* in a phylogenetically calibrated context

**Fig. 2:**
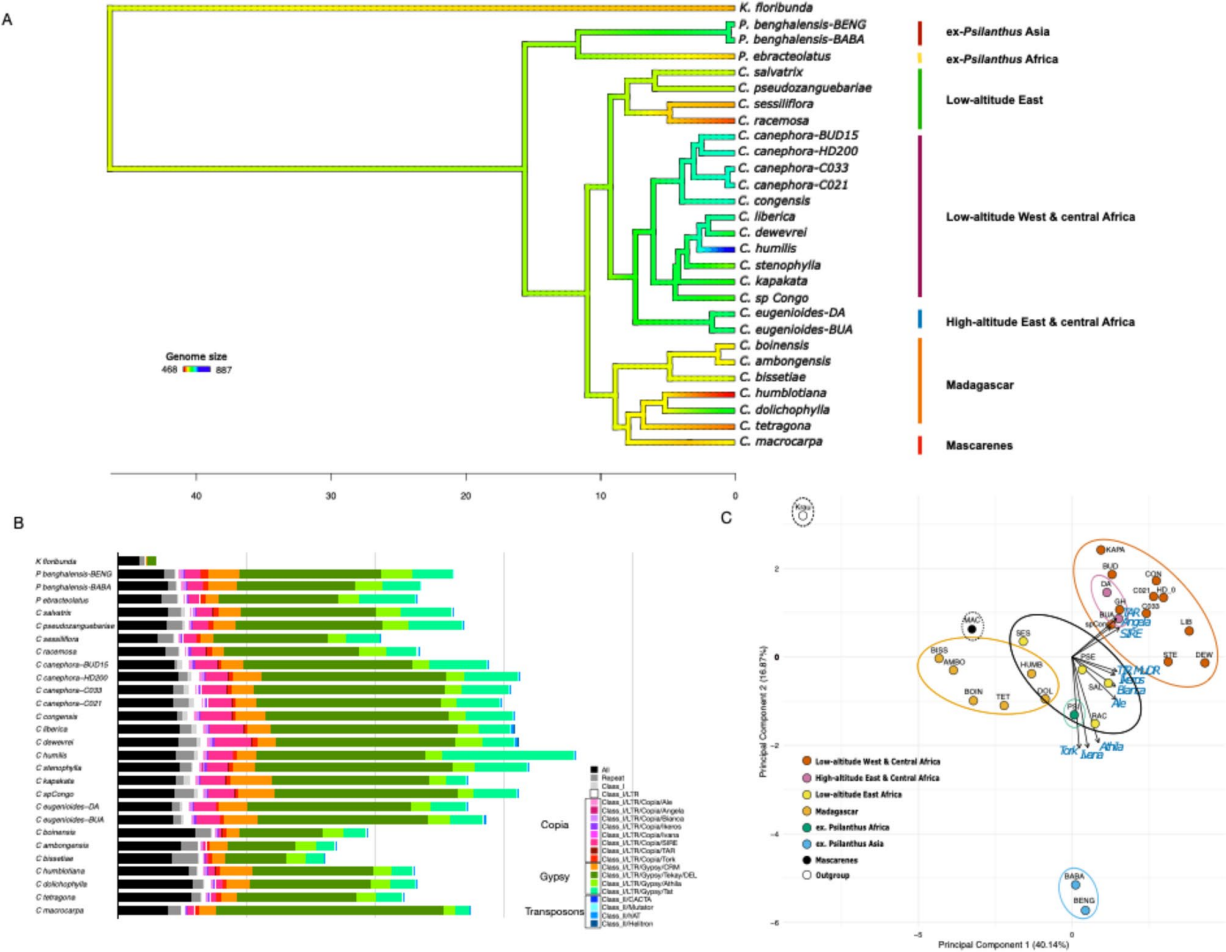
(**A**) Ancestral state reconstruction of genome size. The phylogeographic groups, as defined in^22^, are indicated. Branch colors represent genome size variations (*blue*: large genome size, *red*: small genome size). X-axis represents million years. (**B**) Proportion of identified and unidentified repeats for each accession analyzed using REPEATEXPLORER2 (All: unidentified repeats, Repeat: Identified repeats but still unclassified, Class 1: Identified Retrotransposon but unclassified, Class 1 L: Identified LTR retrotransposons but still unclassified). Each grey vertical bar corresponds to a 20% increment in repeat content. (**C**) Principal Component Analysis (PCA) of accessions based on identified repeats for each accession analyzed using REPEATEXPLORER2 (RAC *C. racemosa*, SAL *C. salvatrix*, SES *C. sessiliflora*, PSE *C. pseudozanguebariae*, KAPA *C. kapakata*, LIB *C. liberica*, DEW C. dewevrei, STE *C. stenophylla*, spCON *C. sp. Congo*, BUD, C033, C021 & HD *C. canephora*, CON *C. congensis*, DA & BUA *C. eugenioides*, HUMB *C. humblotiana*,* DOL C. dolichophylla*, TAT *C. tetragona*, BOIN *C. boinensis*, AMB *C. ambongensis*, BISS *C. bissetiae*, MAC *C. macrocarpa*, BABA & BENG *P. benghalensis*, PSI *P. ebracteolatus*, Krau *K. floribunda*).

Based on short-reads data, either generated in this study or downloaded from NCBI, a total of 28 accessions representing 22 *Coffea* species and one Rubiaceae outgroup species (*Kraussia floribunda*), were aligned against the reference genome of *Coffea canephora*^30^. The same 28,800 SNPs previously used to infer the phylogeny of the genus^22^ were called and utilized to construct a robust and calibrated phylogenetic tree. Ancestral state reconstruction was performed to assess genome size evolution (Fig. 2A; Supplementary Data 2). The analysis showed patterns of genome size variation across *Coffea* lineages. The smallest genomes were observed in the Madagascan clade (*C. humblotiana*, 468 Mb), and lowland East African clade (*C. racemosa*, 499 Mb). The largest genomes were found in the West African lowland clade, with *C. humilis* exhibiting the largest genome size (887 Mb). The divergence between this largest genome (*C. humilis)* and its closest relatives (*C. liberica/C. dewevrei* clade) was estimated at 2.9 million years ago (Mya). A phylogenetic signal analysis for genome size has been undertaken. A moderate phylogenetic signal was detected (Blomberg’s K = 0.5374629). It was supported by the Ornstein-Uhlenbeck (OU) model (α = 0.066775, σ² = 1214.172), suggesting that genome size is partially inherited but that other evolutionary or adaptive forces, in addition to phylogenetic constraints, may have played a significant role in shaping genome size variation within *Coffea*.

### Composition of *Coffea* species in repetitive sequences

The repeat sequence profiles of the 28 accessions analyzed with REPEATEXPLORER2 (identified repeat category) reveal substantial variation in the proportion of repetitive elements within short-read datasets. The repetitive fraction ranged from 24 to 29% of reads in *Coffea ambongensis*,* C. bissetiae*, and *C. boinensis* (Madagascar) to 53% in *C. humilis* (Fig. 2B). Among the LTR retrotransposons, the Tekay lineage (Superfamily *Gypsy*, also called Del) was the most abundant repeat class across all accessions but showed substantial variation from the lowest abundance: *C. bissetiae* (7.04%) to the highest abundance: *C. macrocarpa* (26.4%). Similarly, other LTR retrotransposon lineages showed significant variation like TAT (Superfamily Gypsy, also called Ogre): 1.68% (*C. macrocarpa*) to 15.27% (*C. humilis*) and SIRE (Copia superfamily): 0.2% (*C. bissetiae*) to 4.29% (*C. dewevrei*). A Principal Component Analysis (PCA) performed on phylogeographically grouped accessions and repeat composition data from REPEATEXPLORER2 (Fig. 2C) explained 40.14% (first principal component) and 16.87% (second principal component) of the variance. It also revealed significant separation between the phylogeographic groups: (i) West and Central African lowland species, including *C. canephora*,* C. congensis*,* C. kapakata*,* C. liberica*,* C. dewevrei*,* C. humilis*, and *C. sp. Congo*, with *C. stenophylla* usually found at a slightly higher elevation and drier environment (200 m); (ii) East and Central African highland species, represented by *C. eugenioides*; and (iii) East African lowland species. (*C. racemosa*,* C. pseudozanguebariae*,* C. sessiliflora*,* except C. salvatrix* found between 850 and 1650 m elevation), (iv) the Madagascan species (*C. boinensis*,* C. bissetiae*,* C. ambongensis*,* C. tetragona*,* C. humblotiana*), (v) the Mascarene species (*C. macrocarpa*), (vi) the Asian ex *Psilanthus species* (*P. benghalensis var. bengalensis*,* P. benghalensis var. bababudanii*), and the African ex *Psilanthus species* (*C. ebracteolata*, ex *P. ebracteolatus*). A strong correlation was observed between West and Central African lowland species and several repeat sequence families, particularly the Tekay, TAT, and SIRE lineages. Interestingly, the Asian ex *Psilanthus* group formed a distinct cluster, indicating a highly divergent repeat composition profile. In contrast, the African ex *Psilanthus* accession (*P. ebracteolatus*) was grouped with East African lowland species. The Madagascan group showed distinct repeat composition patterns, except for a partial overlap with the Mascarene group. Together, these results strongly suggest that TE composition profiles can be used to differentiate phylogeographic groups within *Coffea*.

**Fig. 3:**
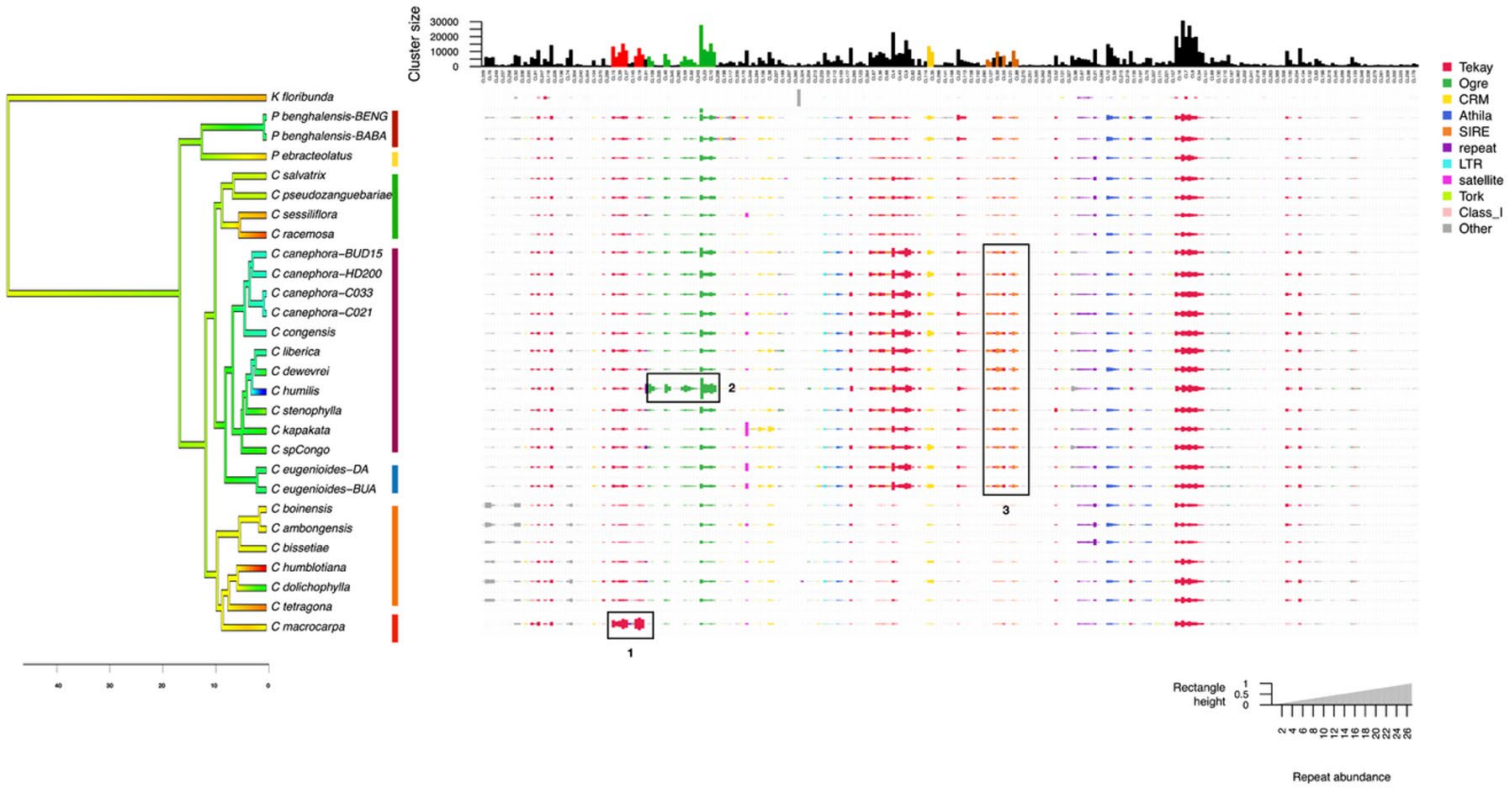
Comparative analysis of repeat profiles in 22 *Coffea* species (28 accessions) and one outgroup, normalized by genome size as estimated by flow cytometry. The top 370 clusters identified by REPEATEXPLORER2 are shown alongside the phylogeny of the species and their respective phylogeographic groups. The bar chart (top) represents the size (number of reads) of individual clusters. The color of the rectangles corresponds to the final annotation of each cluster, and their size is proportional to the abundance of the repeat in each genome.

In total, 2,750,000 short reads were analyzed with REPEATEXPLORER2, of which 1,950,922 reads (70.9%) were grouped into 233,386 clusters. Some 43% of reads were grouped into 370 ‘top clusters’, identified as repetitive elements, including TEs. A comparative analysis of repeatomes (Fig. 3) revealed contrasting profiles among phylogeographic groups and species. The Mascarene Islands species is characterized by several reads associated with the Tekay lineage (Fig. 3, box 1). The west and central African lowland & high-altitude groups displayed high read numbers associated with TAT, Tekay (distinct families from Mascarene), CRM, and SIRE lineages (Fig. 3, box 3). The Madagascar group showed an almost complete absence of SIRE, TAT, and Tekay, with an intermediate profile composition between the East African and West/Central African groups. Interestingly, *C. humilis* displayed a notable increase in the number of TAT lineage reads, which was clearly visible in the analysis (Fig. 3, box 2). To confirm that phylogeographic groups can be differentiated based on their repeat composition, we performed several statistical tests. The PERMANOVA analysis (F-statistic = 13.67, *p* = 0.001, R² = 0.81444) and the Redundancy analysis (RDA, F-statistic = 13.67, *p* = 0.001) suggest that the phylogeographic groups explain a significant portion of the variance in repeat composition. The Kruskal-Wallis test supports that 17 repeat sequence families showed significant abundance differences between phylogeographic groups (*p* < 0.05) (Supplementary Data 3 and 4). The most statistically significant families are the SIRE lineage (*p* = 0.0007), the Tekay lineage (*p* = 0.001), and Class 1 retrotransposons (including LTR and non-LTR retrotransposons, *p* = 0.001).

The repeat sequence profiles identified by REPEATEXPLORER2 were used to assess correlations with genome size (Supplementary Data 5). A significant correlation was found between genome size and the total number of repeated reads (R² = 0.45; *p* = 6.01e-5). Among the LTR retrotransposon lineages, the TAT lineage showed a significant correlation with genome size (R² = 0.43; *p* = 1.26e^− 4^). The SIRE lineage also exhibited a strong correlation (R² = 0.43; *p* = 1e^− 4^). However, the Tekay lineage, showed only a weak correlation (R² = 0.21; *p* = 0.0129). These results suggest that genome size variation can be partially explained by differences in the abundance of repetitive sequences, particularly the TAT and SIRE LTR retrotransposon lineages. Phylogenetic Generalized Least Squares (PGLS) analyses were conducted to understand more precisely the relationships between genome size, Tekay, TAT and SIRE counts while considering the shared evolutionary history of *Coffea*. Results indicate that the quantity of TAT and SIRE counts has a positive and significant effect on genome size, and that the phylogenetic structure has a very strong influence on the data (λ = 1) (Supplementary Data 5 A and B). However, the R² of 43.3% and 16.6% respectively for TAT and SIRE, while not negligible, suggest that other variables or processes might also play an important role in determining genome size. Although the phylogenetic signal is strong (λ = 1), the amount of Tekay doesn’t explain the variation of genome size (Supplementary Data 5 C).

**Fig. 4:**
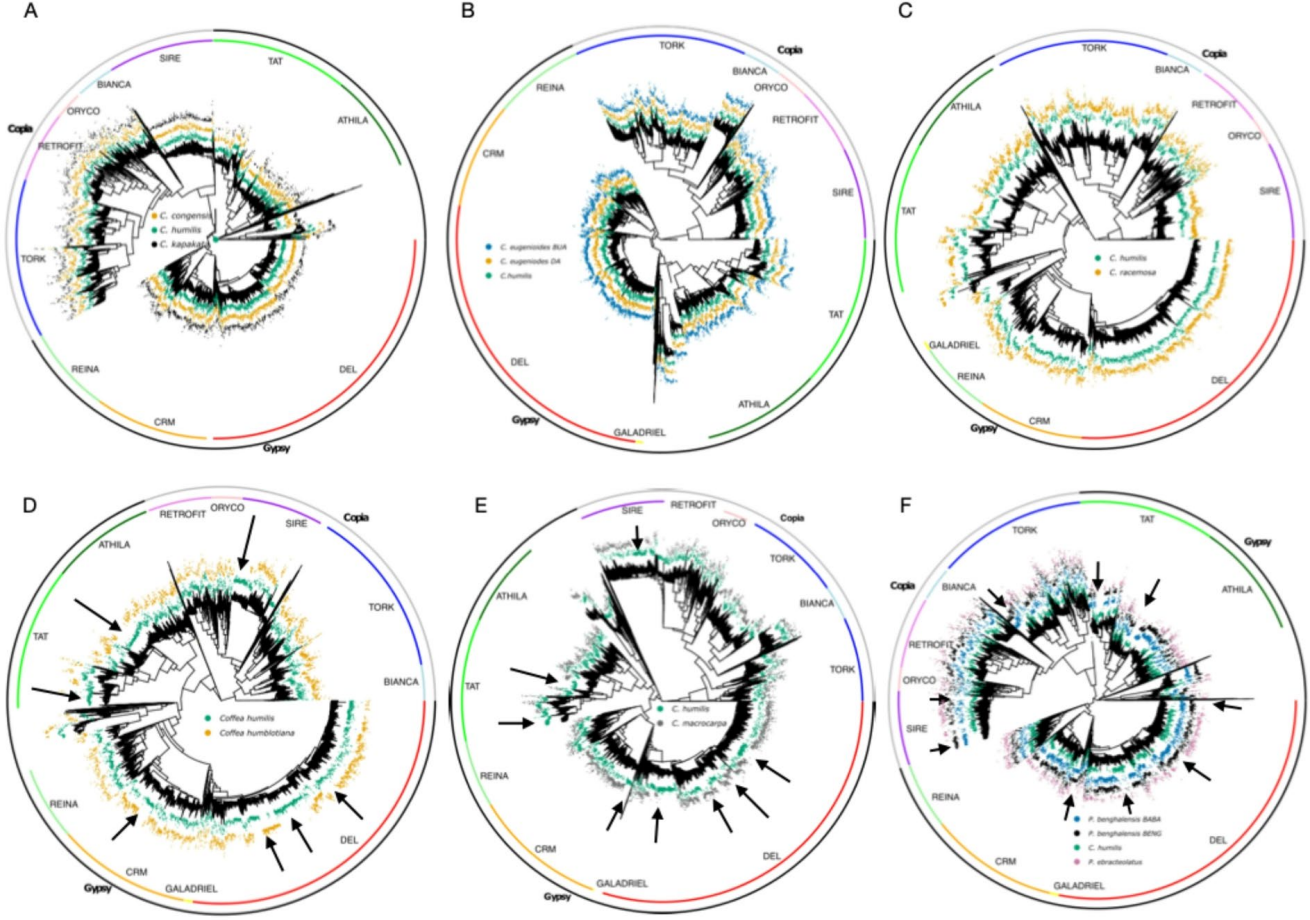
Comparative phylogenetic trees based on Reverse Transcriptase (RT) domains extracted from draft genome assemblies sequenced using short reads. (**A**) Comparison of RT domains in *Coffea humilis*,* C. congensis* and *C. kapakata*. (**B**) *C. humilis* and *C. eugenioides*. (**C**) *C. humilis* and *C. racemosa.* (**D**) *C. humilis* and *C. humblotiana.* (**E**) *C. humilis* and *C. macrocarpa.* (**F**) *C. humilis* and *C. ebracteolatus (*ex *Psilanthus ebracteolata)*, *C. benghalensis* (ex *Psilanthus benghalensis*). var. *benghalensis* and var. *bababudanii*. Colored arrows indicate the expansion of families.

To better visualize the relationships between families composing each lineage of TEs, we conducted a phylogenetic analysis based on the Reverse Transcriptase (RT) domains of LTR retrotransposons identified in *Coffea* genomes assembled from our short reads sequencing data. This comparison aimed to assess the differences between large and small genomes and differences between phylogeographic groups. The phylogenetic trees produced (Fig. 4) were consistent with the REPEATEXPLORER2 results. The comparison between *Coffea humilis* (large genome) and *C. humblotiana* (small genome) (Fig. 4D) revealed that certain families from the Tekay, CRM, TAT, and SIRE lineages were clearly associated with *C. humilis* but absent in *C. humblotiana*. This suggests that genome expansion in *C. humilis* was driven by the amplification of specific LTR retrotransposon families in these lineages. Similarly, distinct LTR retrotransposon families differentiate *C. humilis* and Mascarene species (*C. macrocarpa*) (Fig. 4E), with specific expansions of Tekay elements. Major differences in repeat composition were also observed between *C. humilis* and African and Asian ex *Psilanthus* species (Fig. 4F), reinforcing their genomic divergence. However, no significant differences in repeat composition at this level of analysis were observed between *C. humilis* and lowland East and West African species (Fig. 4A–C), suggesting that the expansion in *C. humilis* is not driven by different repeat families but rather by the differential amplification of existing families.

### Factors influencing genome size and repeatome composition in *Coffea* phylogeographic groups

**Fig. 5:**
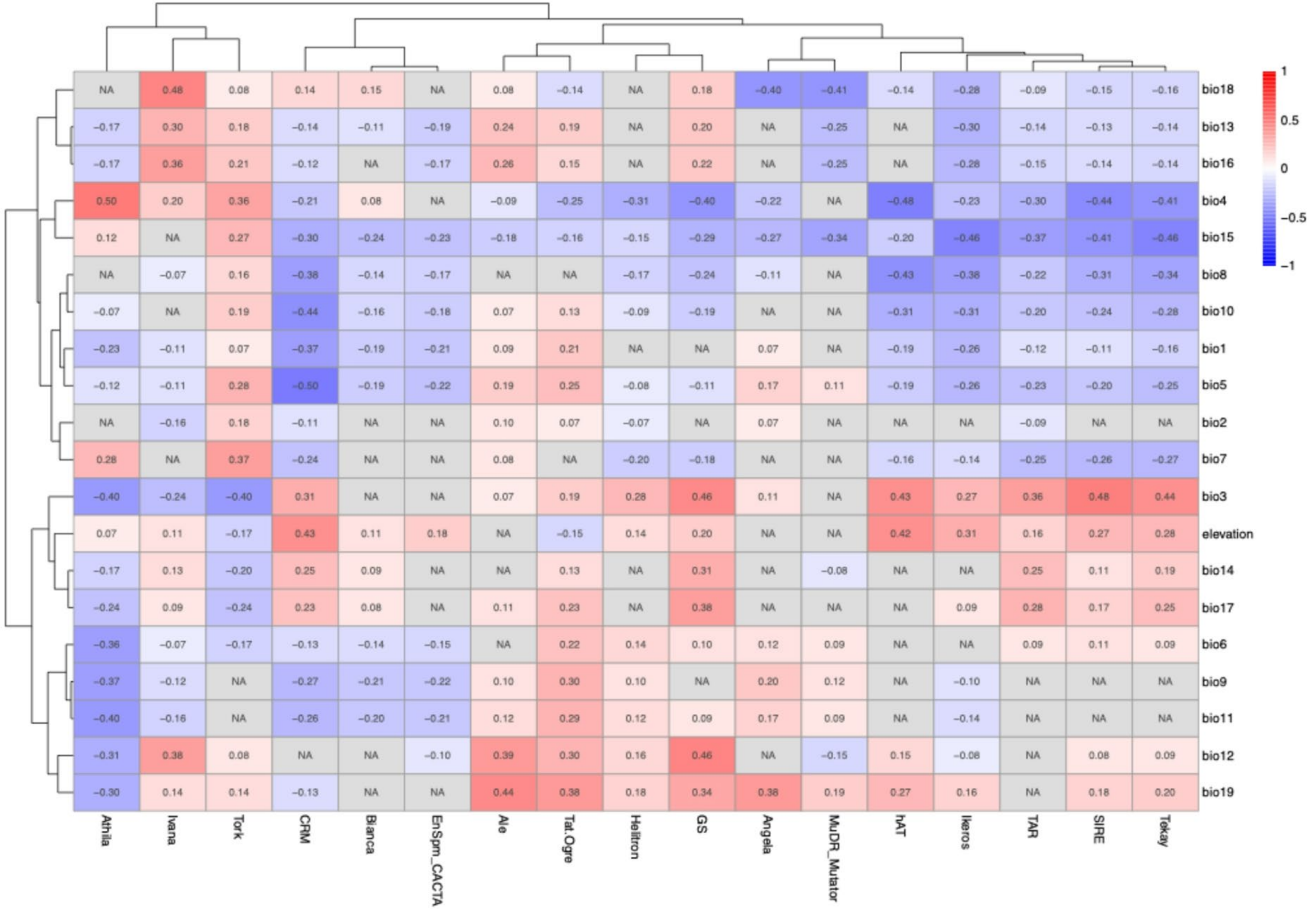
Pearson correlation matrix between TE composition profiles, genome size (GS), elevation and 19 bioclimatic data from WorldClim of *Coffea* species. Correlation analysis of 799 GPS locations from 18 *Coffea* species with their TE profiles identified previously using REPEATEXPLORER2. All correlations are shown if p values ≤ 0.05. NA indicate correlations with p values > 0.05. LTR Retrotransposons Gypsy: Athila, CRM, TAT, Tekay; LTR Retrotransposons Copia: Ivana, Tork, Bianca, Ale, Angela, Ikeros, TAR, SIRE; DNA transposon: EnSpm, Helitron MuDR, hAT.

Since TE composition profiles differentiate *Coffea* phylogeographic groups, we tested whether this accumulation in *Coffea* genomes correlates with environmental variables from the geographic locations of these species. A correlation analysis was conducted between multiple variables: the genome size (GS), the repeat composition (as analyzed via REPEATEXPLORER2), the 19 bioclimatic variables from WorldClim and the elevation data, extracted from the GPS locations of all studied species occurrences (Fig. 5, Supplementary Data 1).

The genome size showed significant positive correlations with the Bio3 variable (Isothermality, *r* = 0.46, *p* = 1.25e^− 43^) and the Bio12 variable (Annual Precipitation, *r* = 0.46, *p* = 9.75e^− 43^). It also showed a significant negative correlation with the Bio4 variable (Temperature Seasonality, *r* = −0.40, *p* = 2.9e^− 33^). For the TE composition, our results showed positive and moderately strong correlations (*r* > 0.4) between Bio3 (Isothermality) and SIRE (Copia retrotransposons, *r* = 0.48, *p* = 3.19e^− 48^), hAT (Class 2 DNA transposons, *r* = 0.43, *p* = 5.83e^− 39^) and Tekay/Del (Gypsy LTR retrotransposons, *r* = 0.44, *p* = 1.06e^− 33^). The temperature seasonality variable (Bio4) showed positive correlations with Athila (Gypsy retrotransposons, *r* = 0.50, *p* = 4.8e^− 47^), the precipitation of the Warmest quarter variable (Bio18) correlated with Ivana (Copia retrotransposons, *r* = 0.48, *p* = 2.97e^− 46^) and the precipitation of the coldest quarter (Bio19) correlated with Ale (Copia retrotransposons, *r* = 0.44, *p* = 4.2e^− 39^). In summary, TEs respond differently to thermal regimes: Athila elements are abundant in environments with strong temperature seasonality, whereas SIRE, Tekay and hAT tend to thrive under more stable thermal conditions. Likewise, TEs exhibit distinct hygrometric patterns: SIRE and Tekay are reduced by strong seasonal shifts in precipitation, while Ivana is more abundant in hot, humid climates and Athila tends to accumulate in less humid environments with pronounced rainfall seasonality. Interestingly, elevation appears to favor CRM and hAT, which are more abundant in high-altitude habitats.

## Discussion

### Genome size variation in *Coffea* and the role of the repeatome

Numerous studies have documented genome size estimates within the *Coffea* genus^17,25,26,28^. In total, 55 out of 141 *Coffea* species/taxa have available flow cytometry data, showing genome sizes ranging from 460 Mb to nearly 900 Mb^18^ (*wildcoffedb.org*). Intraspecific genome size variation has also been observed, notably in *Coffea canephora*, which has the widest geographical distribution in Africa and occupies diverse habitats. Genome size variations from 690 to 730 Mb have been reported depending on the geographical origin of the accession^26^. The origin of these genome size variations in the *Coffea* genus has been explored in the past using 454 sequencing technology^27^. The results suggested a potential role of LTR retrotransposons in genome size variation. However, these early datasets lacked the resolution needed to understand precise mechanisms, as a robust phylogeny of *Coffea* was not yet available, and short-read-based bioinformatics tools were still underdeveloped at the time of publication. In this study, we carefully selected 22 *Coffea* species from different geographic groups, prioritizing accessions available in living collections and covering the observed genome size extremes (minimum and maximum sizes recorded in cytometry). Additionally, we included species with previously sequenced and assembled genomes using long-read technologies (*Coffea canephora*,* C. eugenioides* and *C. humblotiana*^39^ to compare results from short-read and long-read sequencing approaches. Furthermore, these species were selected to establish a robust, time-calibrated phylogeny based on SNPs^22^, allowing us to study genome size variation over evolutionary time scales within a controlled phylogenetic framework. Our results suggest that genome size variation in *Coffea* is first influenced, to some extent, by phylogenetic constraints (Blomberg’s K = 0.5374629). This means that genome size is partially inherited from a common ancestor, following evolutionary history and selection pressures. Since no whole-genome duplication (WGD) events have been recorded in the *Coffea* genus (except for *Coffea arabica*, which was not included in this study), the TE activity in certain lineages has likely played a role in genome size differences, as previously suggested. To investigate the role of TEs, we identified and annotated them using the widely used repeat clustering and annotation approach implemented in REPEATEXPLORER2. The clustering analyses revealed a significant yet moderate correlation between LTR retrotransposons and genome size, particularly with the TAT (Gypsy superfamily, R² = 0.43) and the SIRE lineage (Copia superfamily, R² = 0.43), considering their proportion in the genomes. This pattern is consistent with the view that LTR retrotransposons can drive genome expansion in plants via their replicative transposition mechanism, which includes an RNA intermediate stage. Their bursts of activity can occur in a very short evolutionary time frame, leading to genome size increases^5^. In line with this mechanism, Phylogenetic Generalized Least Squares (PGLS) analyses indicate that the accumulation of TEs such as TAT and SIRE has contributed significantly to genome size increase in *Coffea* and that the phylogenetic structure has a very strong influence on the data.

These TE families (TAT, SIRE) may be purged or retained, depending on species-specific genome dynamics^40^. In this study, we focused on retrotransposon expansion mechanisms, which are better characterized and more accessible with current tools. However, genome size reduction through the elimination of TEs is also a plausible process. These purging mechanisms remain difficult to assess using short-read sequencing data alone and will require, in the future, fully assembled and annotated genomes for proper investigation.

### Divergence of *Coffea* genomes and the role of the repeatome

The repeatome profiles distinguish the *Coffea* phylogeographic groups in Madagascar and Mascarene Island species, Asian ex *Psilanthus species*, East African species, and West African species. Notably, the SIRE, Tekay, and TAT elements distinguish these groups and their species. These LTR elements have shown a recent insertional activity in the sequenced genomes of *C. arabica*,* C. eugenioides*, and *C. canephora*, and their insertion polymorphisms contribute to differentiation among wild, cultivated, and introgressed *C. arabica* accessions^24^. However, in *Coffea humblotiana* (a Comorian species related to the Madagascar diversity group), the SIRE elements are rare with almost no recent activity, confirming REPEATEXPLORER2 results based on short reads^39^. These SIRE elements seem to contribute to species divergence, with lineage-specific families found only in continental African species and absent in Indian Ocean Island species. Their recent activity makes them a key target for studying genome evolution and stress-induced activation in *Coffea* since SIRE elements are also known to influence genome evolution and speciation in other plant models. Originally identified in soybean^41^ and occasionally carrying an *ENV* domain^42^, SIRE are known to be associated with the speciation process in *Citrus*^10^ and to contribute to the genome expansion in *Bomarea edulis* compared to *Alstroemeria longistaminea* (Alstroemeriaceae, order Liliales)^43^.

Tekay is a well-known lineage of LTR retrotransposons in plants. Classified as “chromoviruses” (it carries a chromodomain)^44,45^, this lineage may modulate genome-environment interactions^46,47^. In the *Coffea* genus, it is the most abundant lineage in both large assembled genomes (*C. arabica*,* C. canephora*,* C. eugenioides*) and small assembled genomes (*C. humblotiana*) with mainly insertion in pericentromeric regions^24^. While the Tekay lineage does not appear to be primarily involved in genome size variation mechanisms as indicated by PGLS analysis (and low correlation *R* = 0.21 *p* = 0.01), it plays a remarkable role in the differentiation of phylogeographic groups, with the emergence of families containing a high number of species-specific copies—one group specific to African species and another to species from Mauritius. Like the SIRE lineage, these families seem to be directly associated with speciation processes. As an example, a Tekay subfamily is amplified in species present in Mauritius. This subfamily is rarely found in other *Coffea* species. Although the role of TEs in island adaptation remains debated^48,49^, we cannot exclude their role in island radiation as proposed for *Drosophila* (Drosophilidae, order Diptera) diversification through TE bursts^50^ induced by environmental stress and disruption of epigenetic control. Alternatively, neutral evolutionary processes such as founder effects, genetic drift, and demographic bottlenecks, may also explain such lineage-specific TE profiles possible without adaptative role.

Finally, the elements of the TAT lineage are also significant in the evolution of the *Coffea* genus. They belong to the Gypsy superfamily and are classified among non-chromodomain retrotransposons^51^. Little is known about the TAT lineage, but its large sequence size (ranging from 10 to 21 kb) and the presence of an additional open reading frame that may encode an envelope-like protein (ENV-like) have been reported. In cultivated coffee species, TAT elements exhibit significant insertional activity with a pericentromeric distribution, similar to the Tekay lineage^24^, and they carry a short tandem repeat (82 bp long) named *Coffea_sat11*, which appears to play an important role in chromatin organization around centromeres^52^. This lineage may exhibit significant insertional activity, as evidenced by its rapid amplification over the past million years in the tea genome (*Camellia sinensis;* Theaceae, order Ericales), where it represents 23% of the genome size^53^. A similar amplification is also observed in *C. humilis*, one of the largest genomes in the *Coffea* genus. A sudden amplification event appears to coincide with the divergence of *C. humilis* from *C. liberica* and *C. dewevrei* approximately 2.9 million years ago.

All these findings point toward a model of differential amplification of multiple TE lineages, including SIRE, Tekay, and TAT, with the emergence of specific families within phylogeographic groups or species. The impact of these accumulations on genome structure remains to be explored, but it may vary between lineages, as they are distributed in different chromosomal compartments in completely sequenced genomes (i.e., pericentromeric regions for Tekay and TAT, pericentromeric and distal regions for SIRE). These results indicate that these lineages have exhibited differential activity over time and space, partially associated with phylogenetic and geographic constraints, suggesting a potential role in adaptation and speciation processes.

### Climate modulated genome divergence in *Coffea* within a phylogenetically constrained framework

It has been proposed that climate could affect genome size variations in plants by affecting the activity of TEs, contributing to divergence and speciation mechanisms. Indeed, certain families of TEs may be sensitive to changing and stressful environmental conditions, which could trigger their transpositional activity^54^. However, some types of stress may, on the contrary, limit their expansion, as observed in palms^16^, illustrating the complexity of the relationship between the environment and genome dynamics.

Our analysis in *Coffea* indicates a negative relationship between genome size and climate seasonality and a positive relationship with stable conditions characterized by minimal variations in relative humidity and temperature. A positive correlation between genome size and environments with abundant water availability was also observed, suggesting that larger genomes are more common in such environments, whereas arid conditions may impose constraints on genome expansion, similar to palm genomes^16^.

Importantly, these climatic correlations occur within a strong phylogenetic signal, indicating that shared evolutionary history represent an important determinant of genome size and repeatome composition. Climatic variables appear to participate or modulate the TE dynamics of some lineages. This reflects the contrasting responses of specific TE families. Notably, the LTR retrotransposon lineages such as SIRE, Tekay, TAR, CRM, and the hAT transposon lineages are positively correlated with stable conditions and negatively correlated with unstable conditions. Conversely, other lineages, such as Tork and Athila, exhibit a clear opposite trend, highlighting again the complex relationship between environmental conditions and TE dynamics at this level of analysis. In particular, the TE families SIRE, Tekay and hAT on the one hand, and Athila on the other could be considered as “genomic thermometers,” as their abundance is closely correlated with varying thermal conditions. Similarly, CRM and hAT may serve as genomic indicators of altitude. However, the environmentally associated patterns observed here should not be interpreted as direct evidence of adaptive evolution. Climatic conditions may primarily influence the rate and extent of TE activity, generating insertions, most of which are expected to be neutral or deleterious. Adaptive TE insertions would represent rare outcomes emerging from this increased activity and subsequently retained by selection.

Finally, each TE family responding to a specific climatic profile (mean temperature, temperature amplitude, precipitation, altitude) appears to play a distinct genomic part, forming a responsive “symphony” to the environment.

Overall, our results indicate that genome size and the activity of certain TE lineages appear to be constrained by climate seasonality, reflecting an evolutionary constraint hypothesis: Are unstable environments favoring more compact genomes in *Coffea?* A similar observation was recently reported in studies on *Coffea* species from Madagascar^17^. In Liliaceae and Brassicaceae, genome size evolution is also influenced by climate seasonality, suggesting that this evolutionary model is not restricted to the *Coffea* genus^55,56^.

These findings further emphasize the significant role of the Tekay and SIRE lineages as potential key elements in the genomic response to environmental conditions, influencing genome divergence. Future studies should investigate these lineages in greater detail using evolutionary and adaptation models for *Coffea* species.

Finally, our results highlight the combined influence of phylogeny and environmental factors on the macroevolutionary dynamics of *Coffea* species. More detailed analyses should be conducted in the future using complete genome sequencing, assembly and annotation to better understand the impact of these TE families on chromosome structure and gene function.

## Supplementary Information

Below is the link to the electronic supplementary material.

Sup. Data 1. GPS positions, genome size and bioclimatic data (Worlclim) for the species used in this study. Lat: latitude, long: longitude, group: phylogeographic group, bio1 to bio19 (worldclim data), All to Satellite columns: RepeatExplorer results (number of reads per elements).

Sup. Data 2. Phylogenetic tree of species used in this study with bootstraps.

Supplementary Material 3

Supplementary Material 4

Sup. Data 5. Correlation between genome size (Mb) and all repeated reads.

## Data Availability

The data used in this study is available with bioproject accession numbers PRJEB100521 at Eu-ropean Nucleotide Archive (ENA, EMBL-EBI) and PRJNA898910, PRJNA242989 at Nation-al Center for Biotechnology Information (NCBI).
