# Supplementary figures and images for: Evolutionary history and climate-driven dynamics of transposable elements has shaped genome evolution in the Coffea genus
Source: Sci Rep. 2026 Feb 18;16:9760. doi: 10.1038/s41598-026-40031-6 (PMC13013560; doi:10.1038/s41598-026-40031-6)

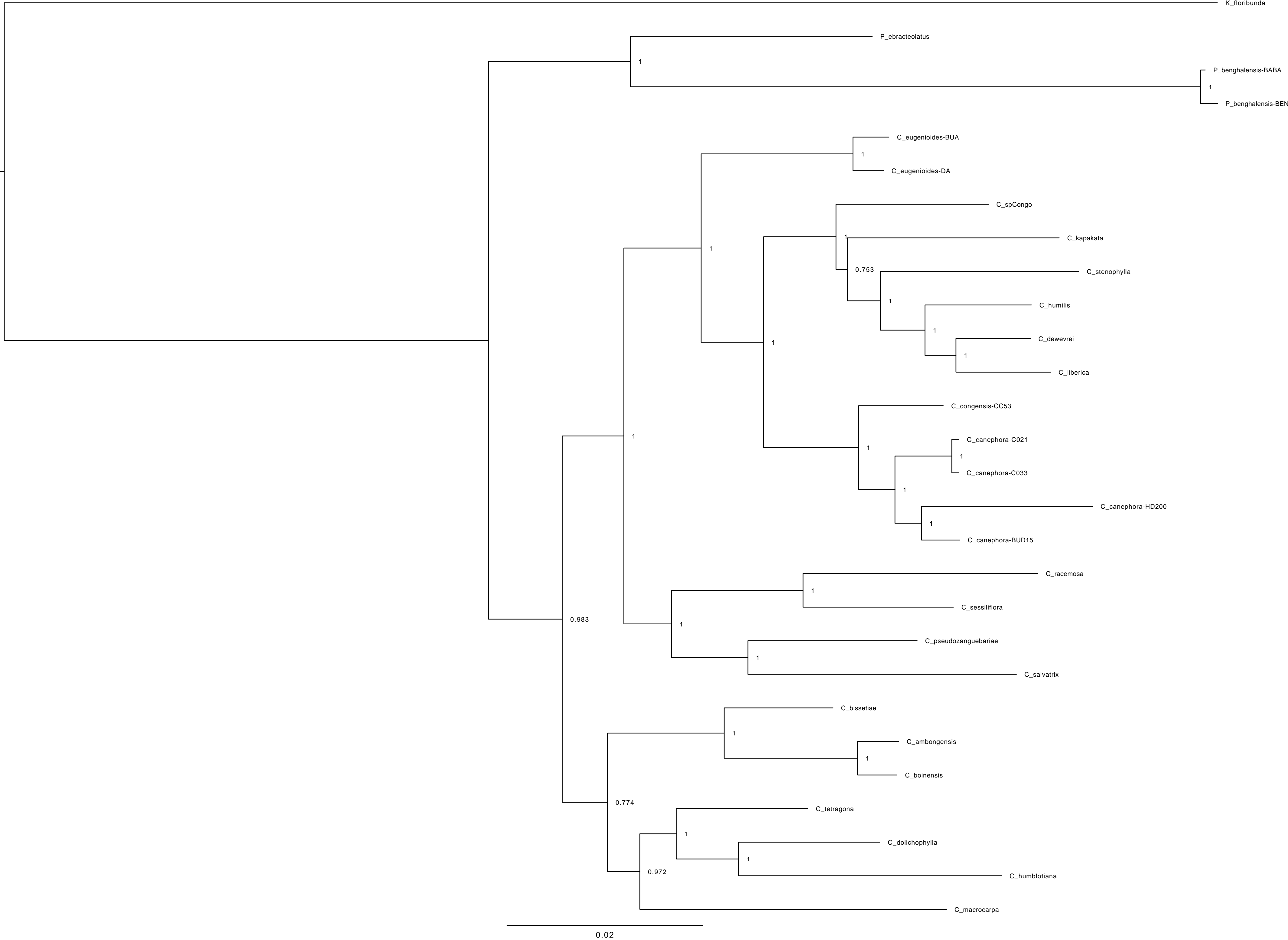

Supplement: Supplementary file 2 — Sup. Data 2. Phylogenetic tree of species used in this study with bootstraps. [file 41598_2026_40031_MOESM2_ESM.pdf]
