# Supplementary material for: Evolutionary history and climate-driven dynamics of transposable elements has shaped genome evolution in the Coffea genus
Source: Sci Rep. 2026 Feb 18;16:9760. doi: 10.1038/s41598-026-40031-6 (PMC13013560; doi:10.1038/s41598-026-40031-6)
Supplement: Supplementary file 3 — Supplementary Material 3 [file 41598_2026_40031_MOESM3_ESM.pdf]

Sup. Data 3. Statistic analyses between phylogeographic groups and the transposable elements composition. A. PERMANOVA. B. Redundancy Analysis (RDA). C. Kruskal-Wallis test

A. PERMANOVA

Permutation test for adonis under reduced model
Terms added sequentially (first to last)
Permutation: free
Number of permutations: 999

	Df	SumOfSqs	R2	F	Pr(>F)
group_phylo	7	1085905311	0.81444	13.167	0.001 ***
Residual	21	247414199	0.18556		
Total	28	1333319510	1.00000		

B. Redundancy Analysis (RDA)

Permutation test for rda under reduced model
Permutation: free
Number of permutations: 999

	Df	Variance	F	Pr(>F)
Model	7	38782333	13.167	0.001 ***
Residual	21	8836221		

C. Kruskal-Wallis test

	Family	p_value	Significance
SIRE	SIRE	0.0006996449	*
Tekay	Tekay	0.0010647542	*
Class_I	Class_I	0.0013659298	*
Ale	Ale	0.0014667193	*
All	All	0.0016031143	*
Helitron	Helitron	0.0018315888	*
Class_I.LTR	Class_I.LTR	0.0028649116	*
Angela	Angela	0.0044465131	*
TIR.MuDR_Mutator	TIR.MuDR_Mutator	0.0059306992	*
TIR.hAT	TIR.hAT	0.0077078448	*
Tat.Ogre	Tat.Ogre	0.0092410437	*
TAR	TAR	0.0093848265	*
Tork	Tork	0.0120026168	*
Athila	Athila	0.0190221927	*
CRM	CRM	0.0237621408	*
Bianca	Bianca	0.0443453794	*
Ikeros	Ikeros	0.0475346561	*
pararetrovirus	pararetrovirus	0.0778634939	ns
Ivana	Ivana	0.1442793222	ns
Satellite	Satellite	0.3067539993	ns
Repeat	Repeat	0.3782538302	ns
TIR.EnSpm_CACTA	TIR.EnSpm_CACTA	0.5513872590	ns
