# Supplementary material for: Evolutionary history and climate-driven dynamics of transposable elements has shaped genome evolution in the Coffea genus
Source: Sci Rep. 2026 Feb 18;16:9760. doi: 10.1038/s41598-026-40031-6 (PMC13013560; doi:10.1038/s41598-026-40031-6)
Supplement: Supplementary file 4 — Supplementary Material 4 [file 41598_2026_40031_MOESM4_ESM.pdf]

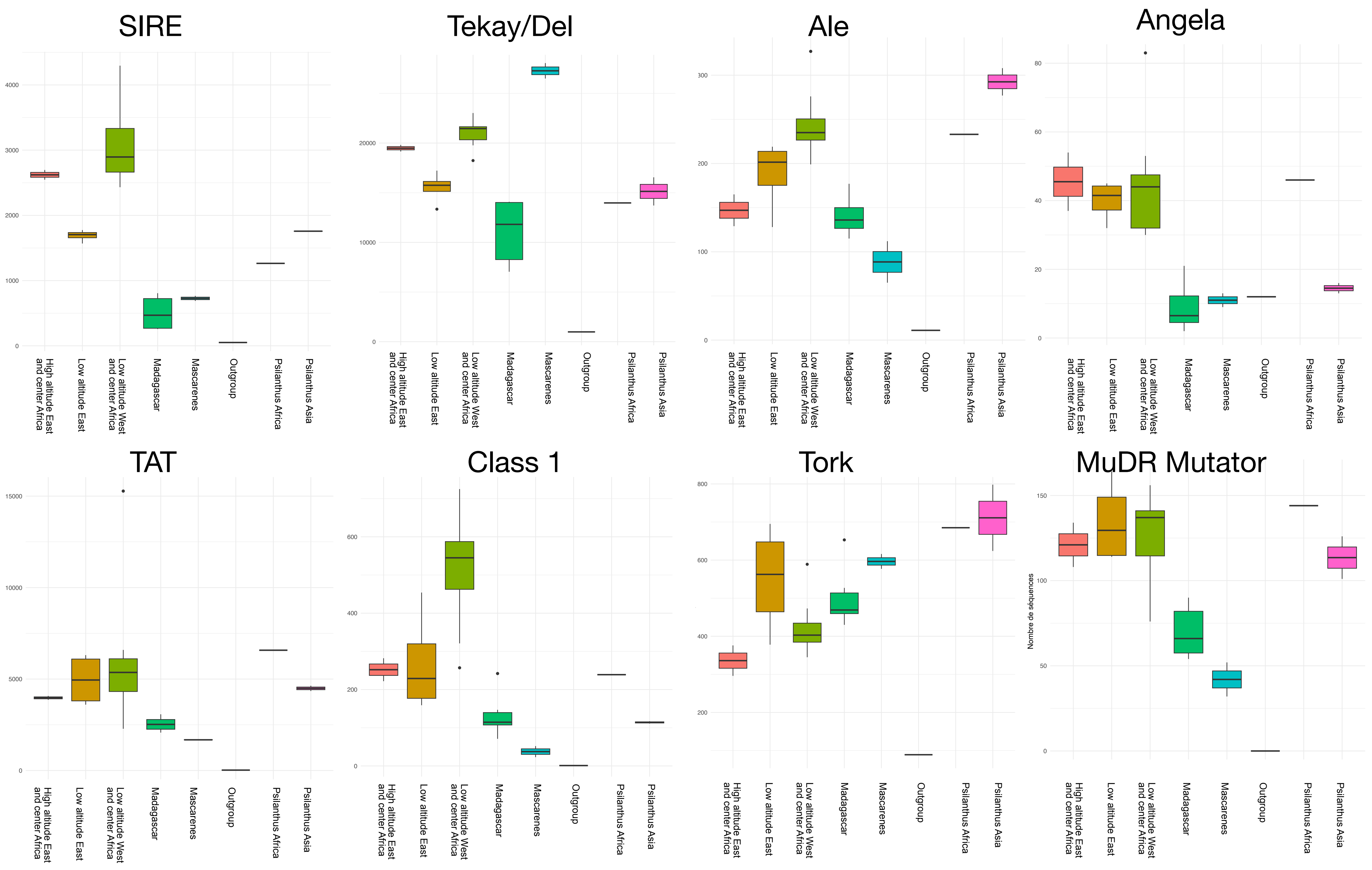

Sup. Data 4. Boxplot of the number of reads for some significant Transposable elements (Kruskal-Wallis test).
