# Supplementary material for: Evolutionary history and climate-driven dynamics of transposable elements has shaped genome evolution in the Coffea genus
Source: Sci Rep. 2026 Feb 18;16:9760. doi: 10.1038/s41598-026-40031-6 (PMC13013560; doi:10.1038/s41598-026-40031-6)
Supplement: Supplementary file 5 — Sup. Data 5. Correlation between genome size (Mb) and all repeated reads. [file 41598_2026_40031_MOESM5_ESM.pdf]

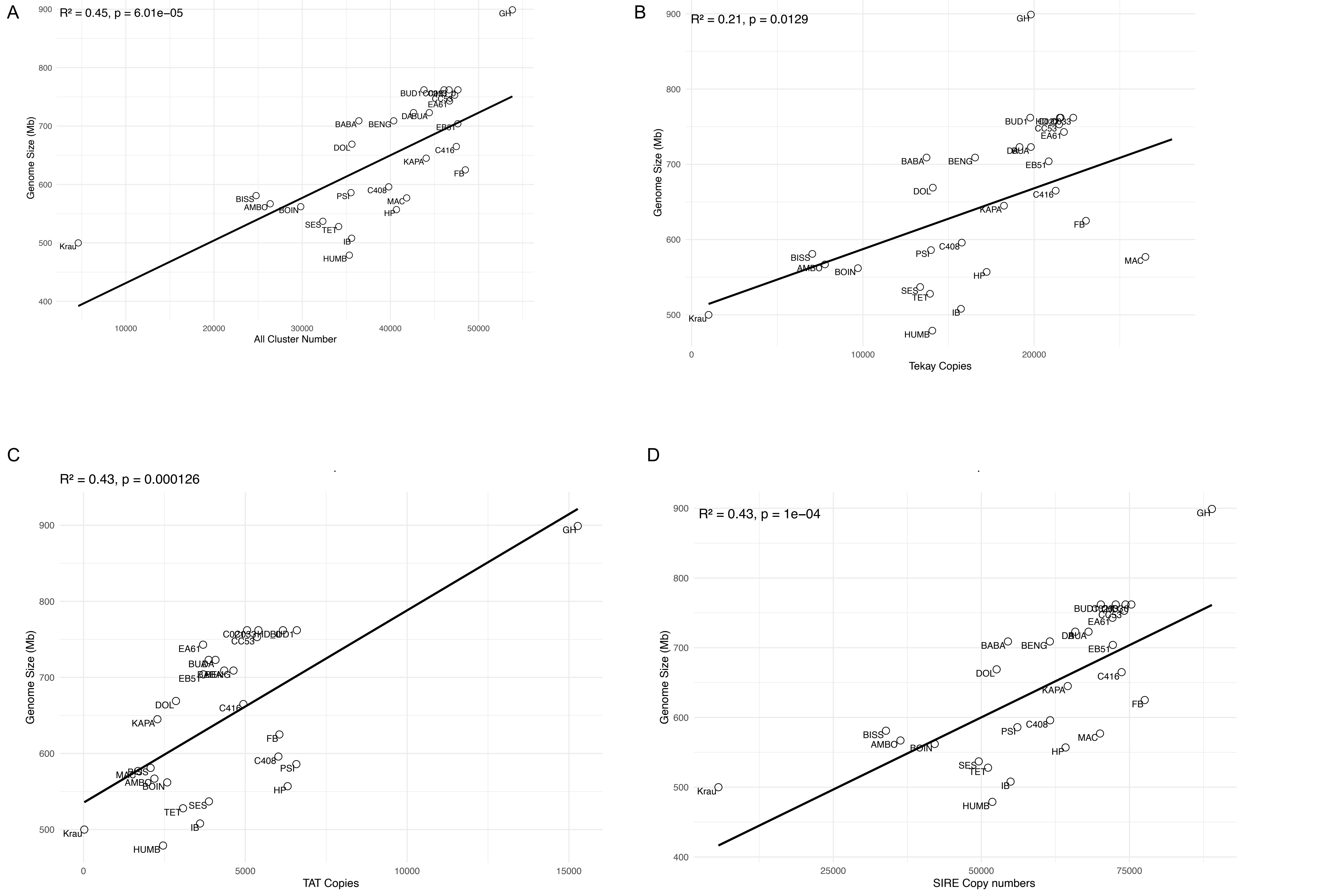

Correlation between genome size (Mb) and all repeated reads (A); LTR retrotransposons Tekay/Del (B); LTR retrotransposons TAT (C) and LTR retrotransposons SIRE (D). Krau: K. floribunda, BISS: C. bissetiae, AMBO: C. ambongensis, BOIN: C. boinensis, SES: C. sessiliflora, TET: C. tetragona, IB: C. racemosa, HUMB: C. humblotiana, PSI: P. ebracteolatus, HP: C. pseudozanguebariae, MAC: C. macrocarpa, C408: C. salvatrix, DOL: C. dolichophylla/C. millotii, BABA: P. benghalensis var bababudanii, BENG: P. benghalensis var bengalhensis, KAPA: C. kapakata, FB: C. stenophylla, C416: C. spCongo, EB51; C. dewevrei, EA61: C. liberica, CC53: C. congensis, BUA: C. eugenioides, DA: C. eugenioides, C021: C. canephora, C033: C. canephora, BUD15: C. canephora, HD: C. canephora, GH: C. humilis,
